# Persistent Circulation of Pseudorabies Virus Variants in China: Genomic Features, Pathogenic Characteristics, and Cross‐Protective Efficacy of Variant Strain Vaccines

**DOI:** 10.1155/tbed/5341313

**Published:** 2026-04-07

**Authors:** Zhenyang Guo, Haonan Kang, Zixuan Feng, Xueli Zhang, Jiahao Shi, Jinhao Li, Ziyu Song, Lirun Xiang, Bangjun Gong, Hu Xu, Chaoliang Leng, Guohui Zhou, Qian Wang, Yandong Tang, Tongqing An, Xuehui Cai, Zhijun Tian, Jinmei Peng, Hongliang Zhang

**Affiliations:** ^1^ State Key Laboratory of Animal Disease Control and Prevention, Harbin Veterinary Research Institute, Chinese Academy of Agricultural Sciences, Harbin, 150001, China, caas.cn; ^2^ College of Veterinary Medicine, Hebei Agricultural University, No. 2596 Lekai South Street, Lianchi District, Baoding, 071000, China, hebau.edu.cn; ^3^ Henan Provincial Engineering and Technology Center of Animal Disease Diagnosis and Integrated Control, Nanyang Normal University, Nanyang, 473061, China, nynu.edu.cn

**Keywords:** cross-protection effect, molecular signatures, novel PRV variants, pathogenicity

## Abstract

The continuous circulation of pseudorabies virus (PRV) variants persists in causing substantial economic losses within China’s swine industry. Nevertheless, the molecular characteristics and pathogenic potential of the recently emerged variants remain poorly understood, and the cross‐protective effectiveness of the newly developed vaccines based on these variants against the circulating strains is still uncertain. In this study, two novel PRV strains, designated WK631 and WK1157, were isolated from clinical samples collected in 2022 and 2024 during investigations of suspected pseudorabies outbreaks. Comparative genomic analysis indicated that both strains share high sequence homology with previously reported PRV variants and harbor sporadic amino acid mutations. Moreover, a small‐fragment recombination event was detected in WK631. Pan‐genomic alignment has identified genotype‐specific molecular signatures: 33 proteins for genotype I, 5 for classical genotype II, and 10 for variant genotype II strains. Subsequently, 14 days after immunization with the PRV variant vaccine, the animals were challenged with WK631, WK1157, or the highly virulent control strain HeN1. Unvaccinated mice exhibited characteristic PRV‐induced pruritus and succumbed to infection with 100% mortality within 6 days postchallenge. High viral loads were detected in brain tissues by quantitative PCR and immunohistochemistry, accompanied by typical neuropathological lesions. In contrast, all vaccinated mice survived without exhibiting any clinical symptoms, viral replication, or pathological alterations. This study not only broadens our understanding of the genomic characteristics of PRV variants but also confirms the pathogenic potential of recent isolates and validates the effectiveness of variant‐based vaccines, thereby reinforcing their potential use in PRV control strategies.

## 1. Introduction

Pseudorabies virus (PRV), classified as Suid alphaherpesvirus 1, is an enveloped double‐stranded DNA virus belonging to the Varicellovirus genus within the Alphaherpesvirinae subfamily [1]. Pigs serve as the only natural reservoir for the virus, facilitating continuous viral carriage and transmission [2]. The primary infection begins in the nasopharyngeal mucosal epithelial cells, subsequently spreading to the peripheral neurons that innervate the area. The virus undergoes retrograde axonal transport to peripheral ganglia, where it establishes lifelong latency [3–5]. Upon reactivation, the virus is transported anterogradely along sensory nerves back to mucosal surfaces, leading to respiratory symptoms in adult pigs and neurological manifestations in piglets [6]. Infection of pregnant sows with PRV can result in abortion or delivery of stillborn or weakened piglets that die shortly after birth [7, 8]. These clinical manifestations have inflicted substantial economic losses on pig production systems. Notably, as a potential zoonotic pathogen, PRV has been associated with severe cases of human encephalitis and endophthalmitis. Recent human PRV infections in China have been correlated with variant strains, highlighting the necessity to characterize these circulating isolates for both animal and public health [9–15].

Phylogenetic analysis based on the gC gene has divided PRV into two genotypes (I and II). Among them, genotype II is predominant in China and can be further classified into two subtypes: classical and variant strains. Classical strains were prevalent in China from the 1960s to the 1970s but were subsequently controlled through widespread use of the Bartha‐K61 vaccine [16]. However, since 2011, PRV has re‐emerged in multiple vaccinated swine herds throughout China. Studies have confirmed that the newly circulating variants exhibited significant antigenic and pathogenic alterations, rendering existing vaccines insufficient for providing complete protection against these emerging strains [17–19]. Since then, PRV variants have spread extensively throughout China, rapidly displacing classical strains and becoming the predominant epidemic lineage [20]. Following the emergence of variant strains in 2011, the seroprevalence of gE antibodies increased significantly, reaching a peak of 39.92% in 2016, while the positivity rate for gE‐specific antigens reached 14.06% during the same period. By 2021, both indicators had decreased. Specifically, the seroprevalence dropped to 15.38%, and the antigen positivity declined to 1.52% [21]. Despite the overall downward trend, certain provinces, such as Henan, continued to exhibit a high prevalence, with an average antigen positivity rate of 7.99% observed between 2021 and 2023 [22]. Moreover, there are notable discrepancies between individual‐ and herd‐level prevalence rates: a 2022 survey conducted across 16 provinces in China reported a serum gE antibody positivity rate of 12.36% at the individual level, compared to 46.22% at the farm level [23]. Notwithstanding the implementation of intensive vaccination programs, the widespread circulation of PRV in China necessitates an investigation into the potential genomic and pathogenic evolution of current strains.

Vaccination has long been recognized as a critical measure for the prevention and control of PRV. Several countries in Europe and North America have successfully eradicated PRV through the implementation of effective DIVA (Differentiating Infected from Vaccinated Animals) strategies [24–26]. The emergence of PRV variant strains in China, and the subsequent absence of specific vaccines, has forced producers to rely on conventional options, including the type I Bartha‐K61 strain and domestically developed type II classical strain vaccines (e.g., SA215, HB98, and HB2000), as their main immunization strategy [27]. These commercial vaccines played a significant role in the early prevention and control of PRV in China, helping to contain large‐scale outbreaks to some extent. However, they have not resolved the persistent circulation of evolving PRV strains in the country. The Bartha‐K61 vaccine has been shown to offer inadequate protection against emerging PRV variant strains, and evidence on the protective efficacy of other currently available vaccines remains limited [17]. However, encouragingly, in 2024, China approved three live attenuated vaccines developed based on PRV variants. These vaccines are expected to more effectively mitigate the spread of circulating PRV variant strains within the country. Nevertheless, given the prolonged development timeline of these vaccines, further evidence is still required to fully evaluate their efficacy against currently prevalent PRV variants.

To investigate the evolving virulence of emerging PRV variants and evaluate the protective efficacy of current vaccines against these strains, whole‐genome sequencing was performed on two wild‐type PRV isolates obtained from vaccinated commercial swine herds between 2022 and 2024. Our objectives are threefold: to characterize the whole‐genome features of these strains and identify molecular markers specific to the variant lineages, to evaluate pathogenicity using murine models, and to assess the protective efficacy of variant‐targeted PRV vaccines. This study provides critical virological and immunological data to inform the development of effective PRV epidemiological control strategies.

## 2. Materials and Methods

### 2.1. Source of Clinical Samples, Viral Strains, Cells, and PCR

Clinical tissue samples were collected from the lungs and brains of infected pigs at swine farms in Heilongjiang Province (2022) and Jilin Province (2024), China, where animals had been vaccinated with Bartha‐K61 yet showed suspected PRV infection. The PRV HeN1 strain (KP098534.1) and Marc‐145 cell line were maintained in our laboratory. DNA extraction and PCR amplification were performed as previously described [19]. Briefly, infected tissues were homogenized by grinding and centrifuged, after which 200 µL of supernatant was collected. Genomic DNA was extracted using a DNA extraction kit (Tiangen, China), and target genes were subsequently amplified using published primers specific to the gB, gE, and gC genes (gBF‐^5`^GTGCTGGCCTCGGACGTCT^3`^, gBR‐^5`^GTTGTAGCGCCGCCGGTAGAT^3`^; gEF‐^5`^ATGCGGCCCTTTCTG^3`^, gER‐^5`^CGGTTCTCCCGGTATTTAAGC^3`^; gCF‐^5`^GACCGTCGCCATGTGTGCCACTAGC^3`^, and gCR‐^5`^ACGCGCGAGAGCCCACACACACACG^3`^) [20]. The PCR products were purified using a gel extraction kit (Tiangen, China), cloned into the pMD18‐T vector (TaKaRa, Japan), and transformed into DH5α competent cells (Tiangen, China). Three positive clones for each fragment were selected and subjected to Sanger sequencing (Kumei, China).

### 2.2. Virus Isolation, Identification, and Growth Kinetics Assay

Virus isolation and purification were performed as previously described [28]. Briefly, tissue homogenates were inoculated onto monolayers of Marc‐145 cells; following the observation of ~70% (24 hpi) cytopathic effect (CPE), viral supernatants from the first passage were collected and subjected to three consecutive rounds of plaque purification. Virus identity was confirmed by indirect immunofluorescence assay (IFA) [29]. Marc‐145 cells were cultured to form confluent monolayers in cell culture plates, inoculated with PRV HeN1 strains, and subsequently monitored for CPE. Upon microscopic observation of CPE, the culture medium was carefully aspirated, and the cells were fixed with prechilled absolute ethanol at 4°C for 1 h. Following ethanol removal and three washes with PBS, the cells were incubated with an anti‐PRV gE monoclonal antibody [30] (37°C, 30 min), after which they were subjected to an additional three PBS washes. The goat antimouse IgG‐FITC conjugate (Sigma, USA) was subsequently applied and incubated at 37°C for 30 min, followed by three washes with PBS. The samples were then resuspended in PBS and examined under an inverted fluorescence microscope for fluorescence visualization. Viral titers were calculated using the Reed‐Muench method and expressed as TCID_50_/0.1 mL. To further characterize viral replication kinetics, a one‐step growth curve analysis was performed: Marc‐145 cells were infected with PRV strains at a multiplicity of infection (MOI) of 0.1, and viral titers were quantified at 2, 4, 6, 12, 18, 24, 30, and 36 h postinfection (hpi).

### 2.3. Whole‐Genome Sequencing of Emerging PRV Variants

Whole genome sequencing was conducted by Shanghai Tanpu Biotechnology Co., Ltd. (Shanghai, China) as part of a commercial service agreement. The procedural workflow was as follows: Viral DNA quantification was initially performed using a biophotometer to evaluate DNA quality based on the A260/280 and A260/230 absorbance ratios. Subsequently, accurate quantification of double‐stranded DNA was carried out using a Qubit 3.0 Fluorometer (Life Technologies, USA) in conjunction with the dsDNA HS Assay Kit (Invitrogen, USA), strictly adhering to the manufacturer’s protocol. The integrity of the purified viral DNA was further confirmed via PCR amplification using the primers specified in Section 2.1.

The Illumina sequencing workflow consisted of four sequential stages: library preparation, cluster generation, sequencing, and data analysis. In brief, viral DNA was fragmented using NEBNext dsDNA Fragmentase. The resulting DNA fragments were processed with the NEBNext Ultra II DNA Library Prep Kit for Illumina (New England Biolabs, USA). Libraries were quantified using the Agilent 2100 Bioanalyzer equipped with the High Sensitivity DNA Kit and subsequently prepared for sequencing on the NovaSeq 6000 platform in accordance with Illumina’s official protocols.

Quality trimming of reads was performed using fastp [31] with additional filtering for adapter sequences and low‐quality reads, including those with a Phred score below Q20. The de novo assembly was conducted in accordance with the metaSPAdes pipeline [32]. The final scaffolds were constrained to a minimum contig length of 100 bases and subjected to a MegaBLAST homology search against the NCBI Nucleotide (NT) database.

### 2.4. Analysis of the Complete Genome Sequence of Emerging PRV Variants

For comprehensive genomic characterization, whole‐genome sequences of 26 PRV strains obtained from the National Center for Biotechnology Information (NCBI) database—including three genotype I, five classical genotype II, and 18 variant genotype II strains—were used as reference genomes for subsequent NT and amino acid sequence analyses. Comparative alignments of the newly identified isolates against the reference strains HeN1, TJ, and Bartha were conducted using the mVISTA platform with the global LAGAN algorithm [33]. Phylogenetic reconstruction was performed based on the gC gene: sequences were aligned using MUSCLE implemented in MEGA version 12 [34], and maximum‐likelihood phylogenetic trees were constructed under the GTR substitution model with support assessed via 1000 bootstrap replicates; the resulting phylogenies were visualized and annotated using the iTOL platform (https://itol.embl.de/). Concurrently, MEGA version 12 was utilized to conduct comparative analyses of NT and amino acid sequence conservation across the target genomes.

To investigate potential recombination events underlying the novel isolates, multiple genome alignments were analyzed using the Recombination Detection Program 4 (RDP4, version 4.101) [35, 36]. Putative recombination signals were screened using seven distinct recombination detection algorithms (RDP, GeneConv, BootScan, MaxChi, Chimera, SiScan, and 3Seq) with Bonferroni correction applied to account for multiple testing. In RDP4, the identification of recombination events requires support from four or more methods [37]. The recombination breakpoints were further refined through analyses using the Genetic Algorithm for SimPlot v3.5.1 [38, 39].

### 2.5. Pathogenicity of Emerging PRV Variants and Protective Efficacy Evaluation of Variant‐Targeted Vaccines

To evaluate the pathogenicity of novel PRV isolates in murine models and to assess the protective efficacy of the commercially available TP strain vaccine (ΔTK/gI/gE/US9/US2 Genes) (Weike, China; Lot: 2025004), derived from PRV variants, against viral challenge, 33 specific pathogen‐free (SPF) ICR mice (female ICR mice aged 6–8 weeks were supplied by Jilin Changsheng Experimental Animal Technology Co., Ltd.) were randomly assigned to seven groups: three mice as blank controls and five mice per group in three TP vaccine‐administered groups (10^4^ TCID_50_/0.1 mL, administered intramuscularly) and five mice per group in three DMEM control groups. 2 weeks after immunization, mice were intramuscularly challenged with 10^4^ TCID_50_/0.1 mL of PRV strains HeN1, WK631, or WK1157. Following the challenge, daily monitoring was performed, with immediate necropsy of moribund mice to aseptically collect cardiac, hepatic, splenic, pulmonary, renal, cerebral, and blood tissue samples. Surviving animals were euthanized at 14 days postchallenge for tissue collection. The collected tissue samples were bisected; one half was fixed in 10% neutral buffered formalin for histopathological examination (H&E staining) and immunohistochemistry (IHC) using an in‐house anti‐PRV gE antibody [30], while the other half was immediately snap‐frozen and stored at −80°C for subsequent viral quantification. Weekly blood collection allowed for the measurement of serum gB/gE antibody titers using a commercial ELISA kit (IDEXX, USA). The criteria are as follows: positive samples: S/*N* ≤ 0.6; negative samples: S/*N* > 0.7; suspicious samples: 0.6 < S/*N* ≤ 0.7. Tissue viral loads were quantified by qPCR using gB/gE‐specific primers/probe (gBF‐^5`^CAAGGTGGACCACAACGTG^3`^; gBR‐^5`^TTGGACAGGAAGGACACCAT^3`^; gBP‐^5`^VIC‐CACGTCGCCGAGGCCCTGGA‐BHQ1^3`^; gEF‐^5`^GAGGTCTGGGACGACCTCTC^3`^; gER‐^5`^CTCGGACACGTTCACCAGAT^3`^; and gEP‐^5`^FAM‐CCGAGGCCGACGACGATGACCTC‐BHQ1^3`^). Viral load calculations were performed in accordance with established methodologies described in previous studies [40].

### 2.6. Statistical Analysis

Statistical analysis was performed using the “Multiple *t*‐tests” approach in GraphPad Prism 8.0 (San Diego, CA, USA). These statistical analyses were applied to the results of virus titration and viral load measurements. Data are presented as mean ± standard deviation (SD). Statistical significance was defined as *p*  < 0.05.

## 3. Results

### 3.1. Virus Isolation, Identification, and Growth Kinetics Assay

Suspected pseudorabies outbreaks occurred in 2022 and 2024 on two commercial swine farms in Jilin and Heilongjiang provinces, both of which were vaccinated with the Bartha‐K61 vaccine. PCR amplification of lung tissues from affected pigs yielded PRV‐specific gB and gE gene fragments, and sequencing results confirmed infection with wild‐type PRV. To characterize the biological properties of these isolates, viruses were propagated in Marc‐145 cells, and the fourth‐passage isolates WK631 (Jilin) and WK1157 (Heilongjiang) were obtained following three rounds of plaque purification. IFA demonstrated both isolates reacted with anti‐PRV gE monoclonal antibodies, exhibiting specific fluorescence (Figure 1A). For the evaluation of growth kinetics, Marc‐145 cells were inoculated with WK631, WK1157, and HeN1 at MOI = 0.1, followed by quantification of viral titers at 2, 4, 6, 12, 18, 24, 30, and 36 hpi. One‐step growth curve analysis revealed that the newly isolated strains exhibited growth kinetics similar to those of the HeN1 strain. Although the HeN1 strain exhibited slightly higher early replication efficiency within the first 6 h postinfection, the difference was not statistically significant. After 6 h, the growth trajectories of all three strains converged and remained similar throughout the subsequent time points. The viral load reached its peak at 30 hpi and then gradually declined. By 36 hpi, the viral titer had stabilized at ~10^7.2^ TCID_50_/0.1 mL (Figure 1B).

Figure 1Biological characteristics of three PRV strains. (A) IFA showing reactivity with PRV gE‐specific monoclonal antibody. (B) One‐step growth curves of the three PRV strains.

**Figure:**
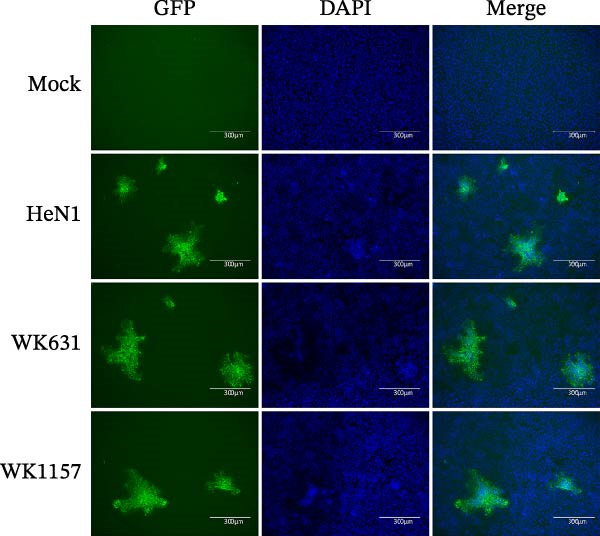
(A)

**Figure:**
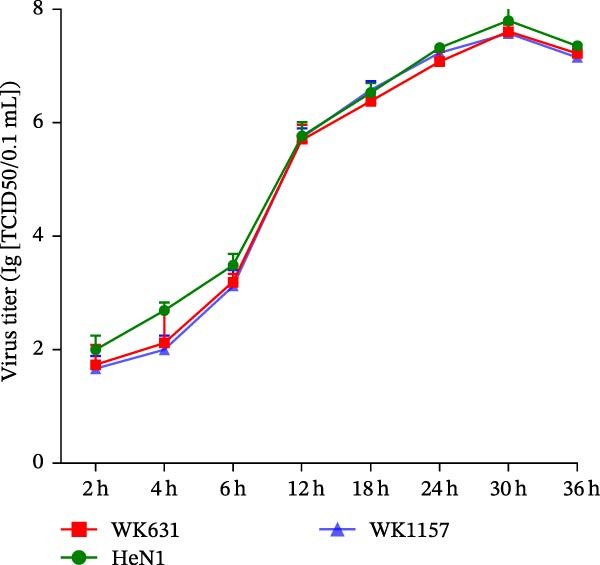
(B)

### 3.2. Whole‐Genome Sequencing and Analysis of Emerging PRV Variants

**Figure 2:**
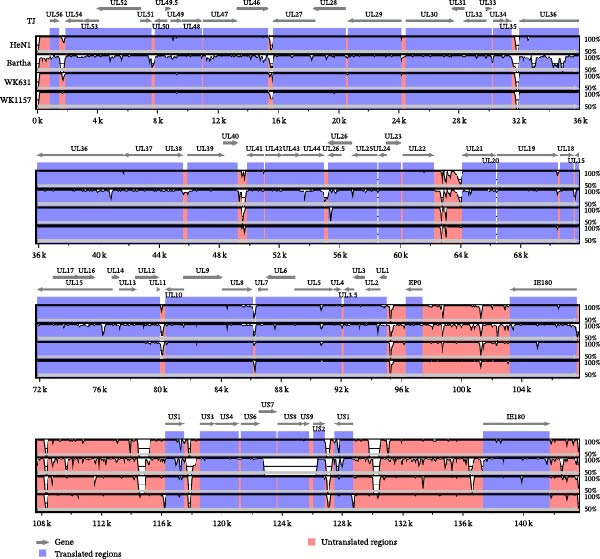
Comparative genomic analysis of complete PRV genome sequences. Sequence similarity analysis among PRV strains HeN1, Bartha, WK631, and WK1157 was conducted with the TJ strain serving as the reference genome. The *x*‐axis represents the genomic position, while the *y*‐axis reflects the percentage of sequence identity. Gray horizontal arrows indicate open reading frames (ORFs). Untranslated regions are highlighted in red, whereas translated regions are shown in blue.

Whole‐genome sequencing of WK631 (GenBank Accession Number PX439453.1) and WK1157 (GenBank Accession Number PX439454.1) revealed genome sizes of ~141 kb and 143 kb, respectively. These novel isolates demonstrated higher sequence similarity to PRV variant strains (HeN1/TJ) compared to the Bartha strain. Notably, WK631 exhibited substantial sequence divergence from TJ in coding regions encompassing UL26, UL25, UL12, UL11, UL10, UL9, IE180, and US1, whereas similarity in noncoding regions was inherently lower (Figure 2). Critically, Bartha exhibited analogous genetic variations at the UL12, UL11, UL10, UL9, IE180, and US1 loci, suggesting that WK631 may have originated from recombination events between the Bartha strain and divergent viral strains. In contrast, WK1157 demonstrated high genomic consistency with TJ (Figure 2).

### 3.3. Recombination Analysis and Coding Gene Characterization of Emerging PRV Variants

To investigate the characteristic mutations in the novel PRV isolates and delineate the molecular signatures that distinguish genotype I, classical genotype II, and variant strains, a comprehensive amino acid sequence alignment of all coding regions from 28 PRV strains was performed. Based on phylogenetic analysis of the gC gene, the 28 PRV strains were classified into distinct evolutionary clades, with the newly identified isolates clustering within the variant branch (Figure 3A). To determine whether WK631 and WK1157 are recombinant viruses derived from PRV variants and the genotype I strain, recombination analysis was conducted using RDP4, with the TJ strain and the Bartha strain serving as reference sequences. Initial RDP4 analysis identified a small recombination event in WK631 spanning the UL12–UL9 genomic region; expanding the analysis to UL13–UL5 (13,900 bp) confirmed recombination via five algorithms, with TJ (variant) as the major parental strain and Bartha‐K61 as the minor parental strain. SimPlot analysis further localized the recombination breakpoints within the UL12, UL10, and UL9 genes (Figure 3B).

Figure 3Phylogenetic and recombination analysis. (A) Phylogenetic analysis based on the gC gene. (B) Recombination analysis of strain WK631. The *x*‐axis represents the genomic position, while the *y*‐axis denotes the percentage of sequence similarity. Recombination breakpoints are indicated by red segments, and blue horizontal arrows located at the bottom of the figure represent gene positions.

**Figure:**
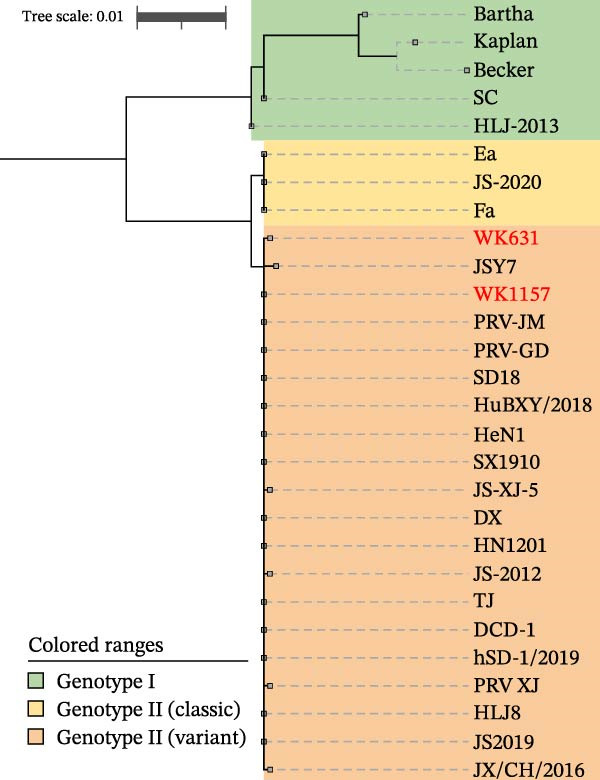
(A)

**Figure:**
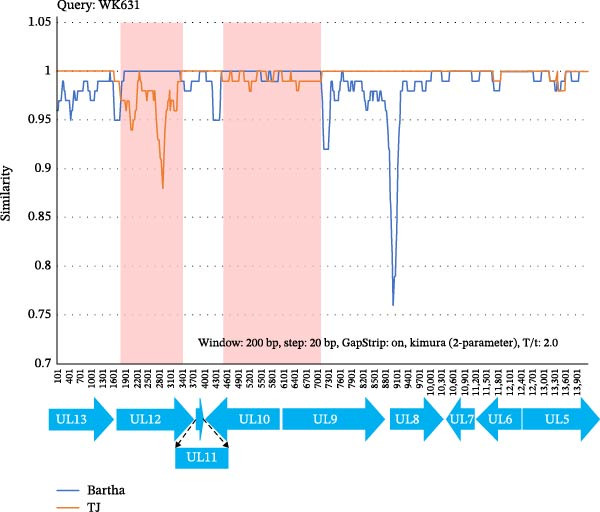
(B)

**Figure 4:**
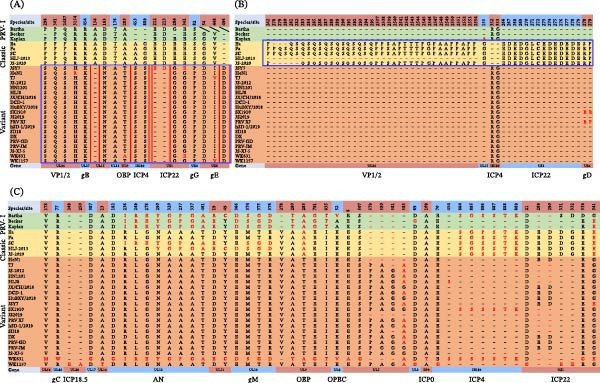
Amino acid alignment analysis. (A) Amino acid‐level conserved mutations in PRV variants. Nonconserved mutations are labeled in red. The solid blue boxes highlight specific mutations present in PRV variant strains, while the solid red boxes indicate pronounced amino acid insertion or deletion mutations (indels). (B) Amino acid‐level conserved mutations in classical PRV strains. Nonconserved mutations are labeled in red. The solid blue boxes denote specific mutations characteristic of classical PRV strains. (C) Amino acid sequence divergence among the novel isolates. Amino acid residues exhibiting divergence are highlighted in red.

Amino acid sequence alignment revealed that, in addition to numerous amino acid substitutions, genotype II strains consistently exhibited insertions or deletions (indels) in 33 viral proteins, including UL52, UL51, UL50, UL49.5, UL49, UL47, UL46, UL27, UL28, UL29, UL36, UL39, UL42, UL44, UL26.5, UL26, UL25, UL21, UL20, UL15, UL17, UL13, UL12, UL9, UL8, UL6, UL3, EP0, IE180, US1, US6, US7, and US8 (Table 1). These indels were predominantly located within the UL region, with the highest density observed in UL36. Furthermore, genome‐wide protein alignment also identified characteristic mutations in PRV variants across nine proteins (Figure 4A): UL36 (P298S, P301Q, Q/P2487H, R3154H), UL27 (R454K), UL15 (154 A^−^, D163N), UL13 (T176A), UL9 (A283T), IE180 (453 S^+^, 889 S^+^), US1 (212‐213ED^−^, E284G, E341G), US4 (S82P), and US8 (G54D, V448I, 496D^+^). Moreover, PRV classical strains exhibited specific insertions or deletions in five proteins (Figure 4B): UL36 (242 P^+^, 278–295PQSQSQSQSQSQSQSQSQ^+^, 2537‐2554PPSAPTTTPGPAAPPAPP^+^), UL15 (155 G^+^), IE180 (352‐353RG^−^), US1 (266‐279EDEDGLCEDEDEDE^+^), and US6 (278‐279RP^+^).

**Table 1:** Compared to genotype I PRV, genotype II PRV exhibits extensive insertion and deletion mutations (indels) in its encoded proteins.

Gene (proteins)	Insertion or deletion sites
UL52	^428^QAHSQ^432^(+), ^596^AL^597^(−), ^622^V (+), ^670^DPE^672^(+), ^680^ *V* (−)
UL51	^182^ *A* (−), ^195^EEA(*V*)DA(*V*)^199^(+), ^206^A (+), ^231^EK^232^(+)
UL50 (dUTPase)	^24^E (+)
UL49.5 (gN)	^4^S (+)
UL49 (VP22)	^34^AVP^36^(+), ^48^DDY^50^(−), ^105^PA^106^(−)
UL47 (VP13/14)	^72^EERM(*T*)S^76^(−), ^126^ *E* (−),^152^ASRAAV^158^(+), ^224^L (+)
UL46 (VP11/12)	^503^PRA^505^(−), ^590^GNAAD^594^(+), ^619^RGSF^622^(−)
UL27 (gB)	^75^SPG^77^(−), ^94^G (+)
UL28 (ICP18.5)	^431^GA^432^(+)
UL29 (ICP8)	^342^G (+), ^1121^G (+),^1140^G (+)
**UL36 (VP1/2)**	^250^GAPAVAAVG^258^(+), ^580^RA^581^(−), ^2288^GRRRRRSR^2296^(+), ^2313^HRNRRRRSSNNSSSSSGG(*R*)SPP^2333^(+), ^2337^ASKK^2340^(+), ^2394^TAPP^2397^(−), ^2419^AQA^2421^(+), ^2427^AATAKPTPQPQPQTQAP^2443^(+), ^2467^ATTAPKA^2473^(−), ^2485^PP^2486^(+), ^2520^PPAPPA^2525^(+), ^2534^QPP^2536^(+), ^2699^S (+), ^2873^A (+), ^2940^AVSA^2943^(+), ^3010^PRAEPARAPAQTRPAPAEP^3028^(+), ^3081^PF^3082^(+), ^3097^SPL^3099^(+), ^3112^E (+), ^3178^P (+)
UL39 (RR1)	^10^SS^11^(−)
UL42	^348^A (+)
UL44 (gC)	^56^AAASTPA^62^(+)
UL26.5 (pre‐VP22a)	^125^PGLP^128^(+), ^209^PA^210^(+)
UL26	^371^PGLP^374^(+), ^455^PA^456^(+)
UL25	^241^A (+), ^317^D (+)
UL21	^365^GGA^367^(+), ^407^APIVS^411^(+)
UL20	^7^V (+), ^19^AAV^21^(+)
UL15	^189^R (+), ^212^GTGSTA^217^(+), ^224^GGG^226^(+), ^1058^RRR^1060^(+), ^1068^ *A* (−), ^1611^PG^1612^(+)
UL17	^252^ *A* (−), ^260^GGG^262^(+)
UL13 (VP18.8)	^27^PGGAIAA^33^(−)
UL12 (AN)	^34^ *A* (−)
UL9 (OBP)	^278^V (+)
UL8 (OPBC)	^125^GGEER^129^(+)
UL6	^8^A (+), ^451^R (+)
UL3	^98^TT^99^(+)
EP0 (ICP0)	^186^ *R* (−)
IE180 (ICP4)	^20^AA^21^(+), ^1425^ *A* (−), ^1441^GAGA^1444^(−)
US1 (ICP22/Rsp40)	^259^EDED^262^(+)
US6 (gD)	^278^R(*S*)P^279^(−)
US7 (gI)	^172^ *H* (−), ^245^T (+)
US8 (gE)	^48^D (+)

*Note:* Superscript Arabic numerals indicate amino acid residue positions; (−) denotes a deletion mutation, (+) denotes an insertion mutation, and italicized text in parentheses (X) indicates the presence of divergent amino acid residues at that position across different strains; Bold text indicates major insertion/deletion hotspots.

The newly isolated PRV strain exhibits a high overall similarity to previously identified PRV variant strains; however, sporadic mutations have already emerged in the amino acid sequences of certain proteins. Comparative analysis revealed that strain WK631 harbored amino acid substitutions in 13 proteins relative to the PRV variants (Figure 4C), including UL56 (V178M), UL44 (R77W), UL17 (D387G), UL12 (D182G, R236I, L249R, G278E, N290Y, A320G, A327P, A337G, T461A), UL11 (D19E, Y49C), UL10 (E366D, MTE374‐376SGD), UL9 (278 V^−^, A280T, T283A, E791G, I835T), UL8 (E52V), UL5 (E8A, S507G, 579‐581PAG^−^, 583A/G), EP0 (A109T), IE180 (454 S^+^, 884‐889SSSSTK^+^), US1 (289‐290ED^−^, E339G, G341E). Notably, the mutations observed in UL12, UL11, UL10, UL9, UL8, UL5, UL3, IE180, and US1 were identical to those present in genotype I strains, underscoring the recombinant origin of this variant from genotype II and genotype I lineages. In contrast, WK1157 closely resembled variant strains, exhibiting only sporadic mutations (Figure 4C), such as in UL28 (249‐250EA^+^), UL16 (A13T), UL3 (D69G), IE180 (E70G), and US1 (D21G, 289‐290ED^−^, 331‐332EE^+^).

### 3.4. Pathogenicity of PRV Isolates in Murine Models and Evaluation of the Protective Efficacy of TP Strain Vaccine

#### 3.4.1. Survival Curves and Viral Load Determination in Mice Post Immunization‐Challenge

To evaluate the pathogenicity of novel PRV variant isolates and the protective efficacy of the commercially available TP strain vaccine against lethal challenge, an immunization‐challenge trial was conducted in ICR mice using field isolates WK631 and WK1157, along with the HeN1 reference strain (Figure 5A). All DMEM‐immunized mice (*n* = 3 groups) succumbed within 6 days postchallenge (Figure 5B), displaying pronounced clinical manifestations of PRV infection, including alopecia in the hip region attributable to self‐scratching (Figure 5C); conversely, all TP‐vaccinated cohorts survived until the end of the trial without exhibiting any clinical sign (Figure 5D). qPCR analysis of major organs, including the heart, liver, spleen, lungs, kidneys, intestines, and brain, revealed high levels of viral DNA—specifically targeting the gB and gE genes—exclusively in the brain tissues of the DMEM control group (no viral antigens were detectable in any other organs). In contrast, no detectable viral signals were observed in the brain or any other organ in either the TP‐vaccinated group or the blank control group (Figure 5E,F).

Figure 5Pathogenicity of emerging PRV variants in mice and protective efficacy of a variant‐based vaccine. (A) Schematic of the immunization‐challenge experiment in mice. (B) Survival curves of mice challenged with different PRV strains. (C) Pruritus‐associated skin lesions in the hip region of a DMEM‐immunized mouse following PRV infection. (D) Absence of lesions in a TP‐vaccinated mouse after PRV challenge. (E) Viral load in brain tissue quantified based on the gB gene. (F) Viral load in brain tissue quantified based on the gE gene. (G) Dynamics of gB‐specific antibody titers following immunization and subsequent challenge. (H) Dynamics of gE‐specific antibody titers following immunization and subsequent challenge.

**Figure:**
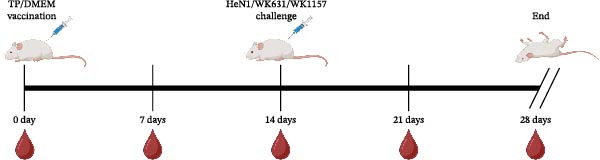
(A)

**Figure:**
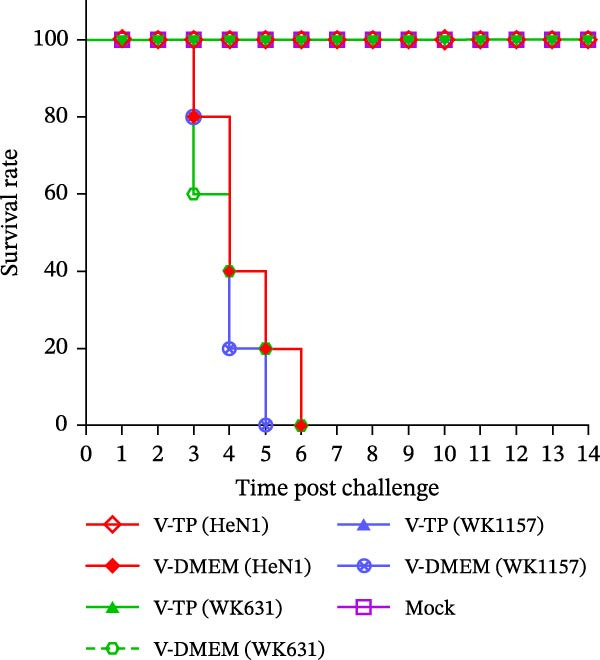
(B)

**Figure:**
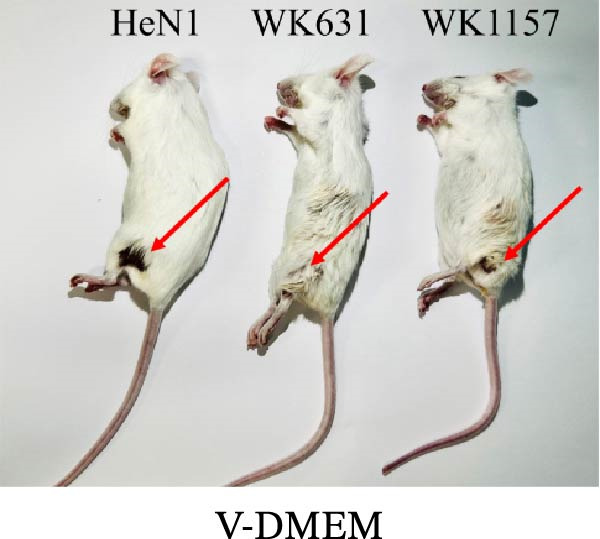
(C)

**Figure:**
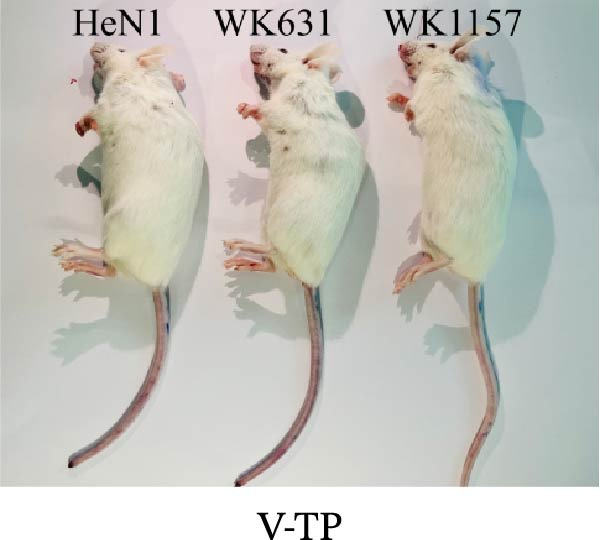
(D)

**Figure:**
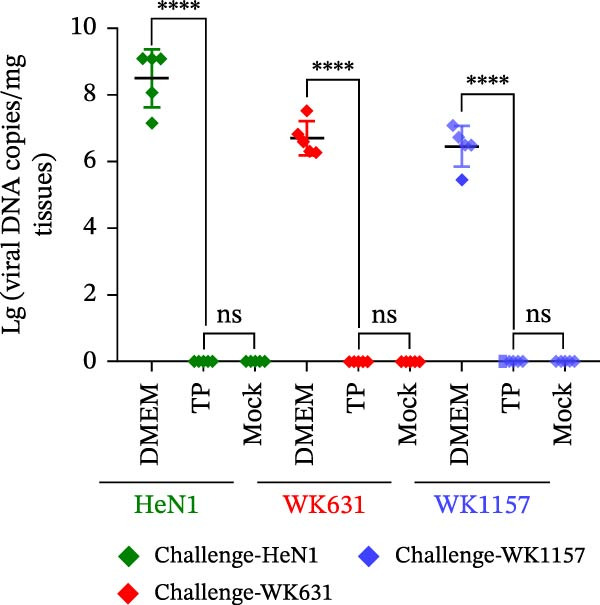
(E)

**Figure:**
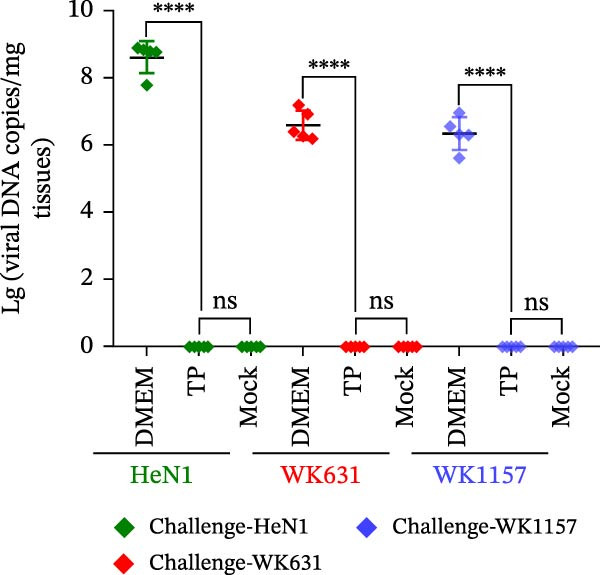
(F)

**Figure:**
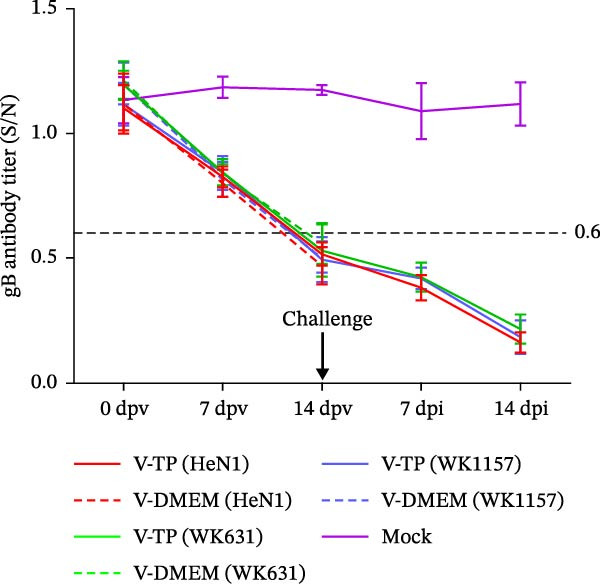
(G)

**Figure:**
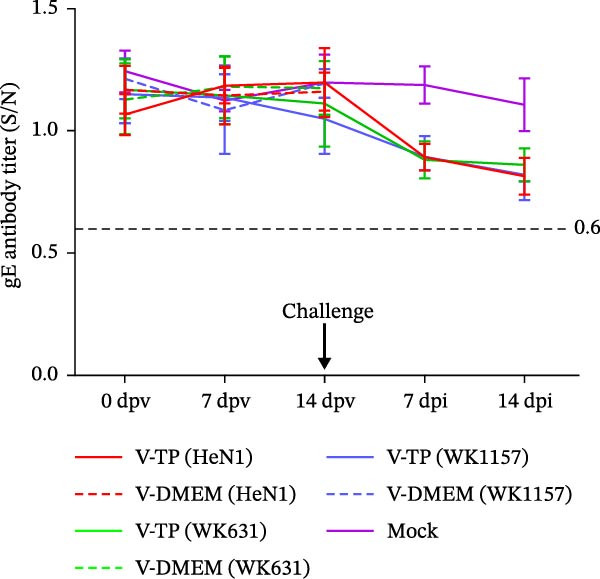
(H)

#### 3.4.2. Dynamics of Serum gB/gE Antibody Titers Postimmunization and Viral Challenge

Serological analysis of PRV gB and gE antibodies during the trial revealed that all TP‐vaccinated mice (*n* = 3 groups) seroconverted for gB antibodies by day 14 postimmunization, with antibody titers showing a progressive increase following viral challenge (Figure 5G); in contrast, gE antibodies were consistently undetectable throughout both the immunization and challenge phases (Figure 5H). The control mice died within 6 days postchallenge, and no postchallenge antibody data were available.

#### 3.4.3. Histopathological Alterations and Immunohistochemical Profiling

Postmortem examinations were conducted on mice that died during the experiment as well as those euthanized upon completion of the study. Brain tissues were collected for histopathological evaluation and immunohistochemical (IHC) analysis. Histopathological analysis revealed characteristic PRV lesions in the brains of all DMEM‐immunized mice following viral challenge. Mice infected with WK631 and WK1157 strains exhibited extensive, diffuse, and sparse glial cell infiltration within both white matter and cortical regions, accompanied by neuronal degeneration and necrosis. Furthermore, the WK631 group exhibited focal spongiform changes in the white matter, whereas the WK1157 group displayed disorganized hippocampal architecture characterized by neuronal swelling, degeneration, and necrosis. In HeN1‐challenged mice, the cerebral cortex exhibited severe and widespread gliosis, marked neuronal necrosis, and mild perivascular hemorrhage in both gray and white matter. In contrast, none of these lesions were observed in the brain tissues of mice from either the TP strain‐immunized group or the blank control group (Figure 6A). IHC analysis consistently detected the presence of PRV gE antigen in the brain tissues of the DMEM‐immunized group, whereas PRV gE antigen was undetectable in both the TP strain‐immunized group and the blank control group (Figure 6B).

Figure 6Histopathological and immunohistochemical examination of mouse brain tissue after immunization‐challenge trial. (A) H&E‐stained horizontal sections of brain tissue. DMEM‐control mice showed extensive, diffuse, and sparse glial cell infiltration in white and cortical matter, neuronal degeneration and necrosis, as well as mild perivascular hemorrhage in gray and white matter (HeN1 challenge); focal spongiform changes in white matter (WK631 challenge); and disorganized hippocampal structure with neuronal swelling, degeneration, and necrosis (WK1157 challenge). (B) Immunohistochemistry was performed using an anti‐PRV gE antibody. PRV antigen was detected in brain tissues of DMEM‐control mice after challenge.

**Figure:**
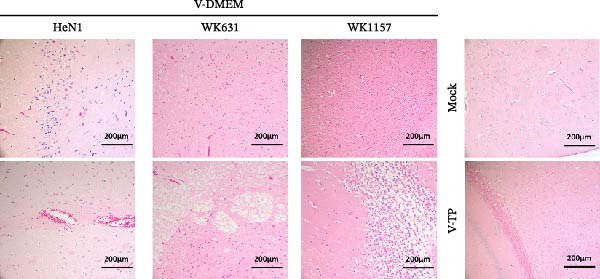
(A)

**Figure:**
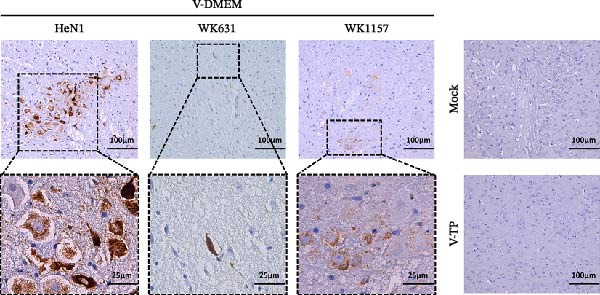
(B)

## 4. Discussion

PRV variant strains have been endemic in China for 14 years, imposing a sustained economic burden on the swine industry. Molecular epidemiological studies contribute to a clearer understanding of viral transmission dynamics; however, it is still uncertain whether currently circulating strains have acquired genomic alterations. Moreover, current knowledge regarding the molecular characteristics of PRV variants is primarily confined to the gE protein as a reference marker, whereas the presence of distinct molecular signatures in other proteins remains to be fully validated. Therefore, this study performed whole‐genome sequencing and immunization‐challenge trials on two PRV field strains isolated in recent years. The molecular characteristics of these isolates were comprehensively analyzed at the whole‐genome level, while their pathogenicity and the efficacy of vaccines specifically designed against variant strains were concurrently evaluated for disease prevention and control. These studies will provide valuable insights into the current epidemiological characteristics, molecular features, and control strategies for PRV.

This study represents the first comprehensive characterization of specific amino acid insertions and deletions across all encoded proteins in genotype II PRV and its classical strains. Overall, the differences between PRV variant strains and classical strains remain relatively limited. On the other hand, the molecular characteristics of PRV variant strains were initially identified by the presence of a “D+” at amino acid position 496 in the gE protein [41, 42]. This signature was subsequently refined to incorporate two distinct patterns: gE aa496 “D+” or aa496 “D−” in combination with aa448 “I” [20]. However, molecular markers in other proteins remained uncharacterized. In this study, whole‐genome sequences of 26 PRV strains representing diverse genotypes were analyzed to compare all coding regions and their corresponding translated amino acid sequences. Characteristic mutations specific to PRV variant strains were identified in the following genes: UL36, UL27, UL15, UL13, UL9, IE180, US1, US4, and US8. Functionally, these genes can be categorized into three distinct groups: viral replication machinery (UL36, UL27, UL15, UL13, and UL9), transcriptional regulation (IE180, US1), and pathogenicity factors (US4, US8) [43, 44]. Whether mutations at these sites serve as the primary drivers of increased virulence in PRV variant strains warrants further experimental validation. Notably, our sequence analysis revealed for the first time that classical PRV strains exhibit characteristic amino acid insertions or deletions in the proteins encoded by the UL36, UL15, IE180, US1, and US2 genes. However, at these same positions, the amino acid sequences of variant strains and genotype I strains remain consistent, indicating a close evolutionary relationship between PRV variants and genotype I strains. We speculate that recombination events may have contributed to this pattern, although the high NT similarity across PRV genotypes likely prevented their detection by recombination analysis software—minor NT variations at these sites may not have been recognized as recombination events. Therefore, we propose that the variant strains associated with the 2011 outbreak in China most likely emerged through a combination of natural evolution and genetic recombination. In this study, whole‐genome sequence analysis was conducted on two isolated PRV field strains. Although both strains belong to the variant strain clade, genomic comparisons revealed distinct differences across their entire genomes. Strain WK1157 largely retains genomic features characteristic of typical variant strains, whereas WK631 exhibits unique mutations in several genes and is highly likely the result of recombination between a variant PRV strain and the Bartha‐K61 vaccine strain. Although this finding indicates recombination events between field isolates and vaccine strains, it does not challenge the well‐established predominance of PRV variants as the predominant circulating lineage.

In herpesviruses, intergenic recombination plays a crucial role in driving viral evolution [45, 46]. Recombination events among PRV strains have been documented since as early as 1993 [47]. The earliest report in China, involving the PRV SC strain, documented recombination between a genotype II classical strain and the Bartha‐K61 vaccine strain, with recombination breakpoints detected in the UL40–UL44 and UL19–UL26 genomic regions [48]. Subsequently, recombinant viruses involving PRV variant strains and Bartha‐K61 were reported, including JSY13, GXLB‐2015, and HuB20 [40, 42, 49]. Additionally, recombination between the HB98 vaccine strain and genotype II classical PRV strains was confirmed [50, 51]. In addition to recombination events between vaccine and field strains, recombination among field strains has also been observed, including between variant and classical strains as well as among classical strains themselves [25, 52]. Recombination sites in PRV are not fixed and may occur in both coding and noncoding regions [52], indicating the absence of defined recombination hotspots. With regard to pathogenicity, the majority of recombinant viruses display virulence levels that are either comparable to or moderately reduced relative to wild‐type strains. Notable exceptions are HeN21 and HuB20, which exhibited increased pathogenicity in adult pigs, leading to the development of respiratory and neurological symptoms [40]. Based on these observations, we speculate that recombination between PRV strains may not significantly enhance viral pathogenicity, although this possibility could depend on specific recombination breakpoints. For instance, in the present study, recombination in the UL12–UL9 region (replication‐related genes) of WK631 did not alter virulence, potentially because this breakpoint region does not encompass key virulence factors such as gE/gI or TK. These findings warrant further investigation for confirmation. Attenuated PRV vaccines are generally regarded as safe, given that postvaccination surveillance typically fails to detect the virus in major organs of immunized pigs. Although recombinant strains were identified in this study, the available evidence suggests that recombination has a limited effect on PRV virulence. The newly developed attenuated vaccine, based on PRV variant strains, conferred complete protection in mice against lethal challenge while remaining compatible with established differential diagnostic approaches. Nevertheless, sustained vigilance is crucial to monitor the potential emergence of highly pathogenic PRV strains through recombination events. Consequently, continuous epidemiological surveillance of PRV should be upheld, with systematic monitoring of genomic evolution in circulating viral populations.

In this study, both wild‐type viral strains exhibited in vitro replication capabilities comparable to those of the early prevalent PRV HeN1 strain. Pathogenicity evaluation demonstrated that following intramuscular inoculation with 10^4^ TCID_50_/0.1 mL of the virus, all mice across the three experimental groups succumbed to infection within 6 days, indicating that the virulence of the newly isolated viral strains in murine models is comparable to that of the previously characterized highly pathogenic strain HeN1. Similarly, qPCR and IHC consistently detected high levels of PRV antigens in brain tissues. Histopathological analysis revealed that viral infection induced widespread neurological damage and cellular apoptosis. Collectively, these findings demonstrate that the currently circulating PRV strains retain virulence levels comparable to those of previously prevalent variants.

As a highly susceptible species to PRV, mice infected with a virulent strain exhibit elevated levels of IL‐6 and G‐CSF, which induce a systemic inflammatory response leading to severe pruritus and acute mortality [53]. Bartha‐K61, for example, can induce fatal infections in mice within 2 weeks, accompanied by severe central nervous system dysfunction. High titers of infectious virus are consistently detected in the brain at the time of death; however, neither pruritus nor cutaneous lesions are observed during the course of infection [54]. In contrast, the TP strain, an attenuated vaccine developed based on variant strains, did not elicit adverse effects in immunized mice in the present study. Instead, it induced a robust immune response that conferred complete protection against lethal challenge with the wild‐type virus. The high safety profile of the TP strain in mice may be attributed to the additional deletion of the TK gene, in conjunction with the prior removal of the gI, gE, US9, and US2 genes. The TK gene encodes an enzymatic protein that is essential for viral replication in neuronal cells. Even a single amino acid substitution at position 13 can abolish its enzymatic activity, resulting in significant viral attenuation in murine models [55–57]. Therefore, we propose that deletion of the TK gene in PRV vaccines could significantly improve their safety profile. Furthermore, although serum gB antibody titers only slightly exceeded the threshold 2 weeks after immunization with the TP strain, the mice were already fully protected against viral challenge. This outcome was unexpected, given that the majority of PRV vaccine studies in murine models include a booster immunization prior to pathogen challenge [58–60]. This finding suggests that the early‐stage protection conferred by the TP strain following vaccination may be predominantly mediated through cell‐mediated immune responses. Previous studies have demonstrated that attenuated PRV vaccines can induce robust cytokine production, including elevated levels of IFN‐γ, IL‐2, and IL‐4, and effectively promote the activation of CD8^+^, CD69^+^, CD3^+^, and CD4^+^ tissue‐resident memory T cells in murine models [58, 59]. Thus, the high efficacy of attenuated PRV vaccines in practical applications may be attributed to their capacity to elicit robust cellular and humoral immune responses, thereby differentiating them from attenuated PRRSV vaccines, which predominantly depend on cellular immunity for pathogen clearance. In conclusion, we propose that vaccines developed based on PRV variants constitute a pivotal component of current PRV control strategies, offering significant advantages in terms of both safety and efficacy. While the murine model demonstrates complete protection, future studies in swine are needed to confirm the TP vaccine’s efficacy in reducing viral shedding and transmission, a critical endpoint for PRV eradication programs.

## 5. Conclusion

In summary, this study performed whole‐genome sequencing of two newly isolated porcine PRV field strains, identifying a recombination event between variant strains and the Bartha‐K61 vaccine strain within the UL12–UL9 genomic region. Furthermore, comprehensive genomic analyses were conducted to characterize the molecular features of PRV across genotype I, genotype II, classical genotype II strains, and variant strains. Immunization‐challenge experiments in mice demonstrated that both isolated strains exhibit high pathogenicity comparable to previously predominant variants. Furthermore, the vaccine specifically developed for these variants provided complete protection against lethal PRV challenge in mice. These findings highlight the importance of continuous genomic surveillance and variant‐specific vaccine development for PRV, a transboundary pathogen with implications for swine industries worldwide.

## Author Contributions

Zhenyang Guo and Hongliang Zhang designed and managed the study. Zhenyang Guo, Haonan Kang, Zixuan Feng, Xueli Zhang, Jiahao Shi, Ziyu Song, Jinhao Li, Lirun Xiang, Bangjun Gong, and Hu Xu conducted the experiments. Zhenyang Guo, Haonan Kang, Zixuan Feng, and Xueli Zhang analyzed the data. Lirun Xiang, Chaoliang Leng, Guohui Zhou, Qian Wang, Tongqing An, and Xuehui Cai collect samples. Zhenyang Guo and Haonan Kang wrote the manuscript. Zhijun Tian, Jinmei Peng, and Hongliang Zhang reviewed and edited the paper.

## Funding

This study was supported by the Key Research and Development Program of Heilongjiang (Grant 2022ZX02B13), the National Key Research and Development Program of China (Grant 2022YFD1800802), and the National Center of Technology Innovation for Pigs (Grant NCTIP‐XD/C09).

## Disclosure

All authors have read and agreed to the published version of the manuscript.

## Ethics Statement

The animal experiments in this study were conducted in the Animal Experiment Center (ABSL‐2) of the Harbin Veterinary Research Institute, Chinese Academy of Agricultural Sciences (Approval Number SYXK (Hei) 2022‐005), approved by the Animal Ethics Committee of the Harbin Veterinary Research Institute (Approval Number 250514‐01‐GR), and performed in strict accordance with the applicable ethical guidelines.

## Conflicts of Interest

The authors declare no conflicts of interest.

## Data Availability

The data that support the findings of this study are directly available in ScienceDB. Research Data at https://doi.org/10.57760/sciencedb.29734.
